# Dietary Treatment Options for Depression among Diabetic Patient, Focusing on Macronutrients

**DOI:** 10.1155/2013/421832

**Published:** 2013-09-30

**Authors:** Fahimeh Haghighatdoost, Leila Azadbakht

**Affiliations:** ^1^Food Security Research Center, Isfahan University of Medical Sciences, Isfahan 21871, Iran; ^2^Department of Community Nutrition, School of Nutrition and Food Science, Isfahan University of Medical Sciences, Isfahan 21871, Iran

## Abstract

There is a bidirectional adverse association between diabetes and depression. The odds for experiencing depressive symptoms in diabetic patients are two times more than nondiabetic persons, and depression is an independent predictor for the onset of diabetes. However, depression has been approximately unrecognized and untreated in two-thirds of diabetic patients, which may lead to worsened diabetes complications. A cornerstone strategy for managing depression among diabetic patients is the use of diet to improve both health problems. Because of similar pathophysiology for chronic diseases and depression, it seems that similar dietary recommendations could be useful. However, few studies have been conducted among diabetic patients. Regarding the complications of diabetes such as renal diseases and coronary heart diseases, the proper range of various macronutrients should be clarified in depressed diabetic patients as well as the proper type of each macronutrient. In this paper, we reviewed the available data on the treatment of depression in diabetic patients.

## 1. Introduction

Major depression is a high prevalent comorbid in patients with type 1 or 2 diabetes [1, 2]. The odds for experiencing depressive symptoms in diabetic patients is two times more than nondiabetic persons, independent of sex, type of diabetes, subject source, or assessment method for depression [1]. Furthermore, the prevalence of depression is higher among diabetic women than men (28% versus 18%) [1]. Depression is considered as an independent predictor for the onset of diabetes [3, 4]. Conversely, the presence of a depressive history during lifetime increases the risk of diabetes in later-life cycle [5–9], and there is a bidirectional adverse association between diabetes and depression. 

The exact mechanisms underlying the relationship between depression and diabetes have not been established yet. Suggested mechanisms by which anxiety and depression increased the risk of diabetes are alterations in insulin signaling in the brain, activation of proinflammatory pathways, and/or distress-induced upregulation of counterregulatory hormone systems like glucocorticoid [10, 11], which could impair insulin sensitivity. Another possible mechanism may be related to the effect of depression on behaviors and lifestyle. It has been shown that depressed persons are more likely to be physically inactive and central and/or general obese and have unhealthy eating habits, poor diet, and sedentary lifestyle [12–15], which is associated with increased risk of coronary heart diseases and diabetes. Elevated serum C-reactive protein (CRP), homocysteine, and higher 24-hour urine free cortisol may provide another additional mechanism whereby depression increases the risk of chronic disease such as CHD and diabetes [16–20]. 

Despite the high prevalence rate of depression among diabetic patients, it has been approximately unrecognized and untreated in two-thirds of diabetic patients [21]. Additionally, because of the severity and chronic course of depression in diabetic patients, 80% of them may experience depression relapse even after successful treatment [22]. Once chronic medical illness appears, comorbid depression increases the burden of symptoms and functional disability [23, 24]. Untreated depression is associated with worsened diabetes complications such as retinopathy, nephropathy, neuropathy, sexual dysfunction and coronary heart disease [25, 26], poorer glycemic control [27], and higher insulin level [28]. Furthermore, depressed diabetic patients were less tended to adhere dietary recommendations, physical activity program, self-care, and medication regimens [29–31]. Depression has some other disadvantage for diabetic patients: the costs of medical therapies are 4.5 times higher for depressed diabetic than nondepressed [31], the odds of experiencing functional disability are 7.15 times higher among depressed-diabetics than nondepressed ones [32], and depressed-diabetic patients have 2.3 times higher risk for mortality compared with diabetic patients without depression [33]. It has been shown that 54% of patients with both problems had early mortality [34]. Therefore, the important health outcomes of depression in diabetic patients necessitate the optimal medical care to improve both health problems. It has been suggested that if glycemic control was not achieved by routine medical therapies, depression should be noted as a potential cause [35].

A cornerstone strategy for managing depression among diabetic patients is the use of diet to improve both health problems. Although the association of dietary intake and depression is a novel field which emerged during last ten years, the similarity between the pathophysiology of chronic diseases and depression may need some similar dietary recommendations. Most of available studies have been conducted among depressed patients without diabetes, and few studies have been conducted among diabetic patients. In this paper, we aimed to review the present studies regarding the dietary approach for treating depression among diabetic patients.

## 2. Methods 

In order to search the relevant English and non-English published papers we used the online databases of PubMed, ISI Web of Science, SCOPUS, Science Direct, and EMBASE for the period from January 2000 to May 2013. The keywords used in our search strategy were depression, depressive, depressed, diabetes mellitus, diabetic patients, diet, macronutrients, fat, protein, carbohydrate, nutrition, and food. We found 410 papers which is published between 1965 and 2013, but some of them were excluded because they were not conducted among diabetic patients or they were not related to dietary intakes especially macronutrients. We found only 7 studies which conducted among depressed and diabetic patients. Two studies assessed the effect of protein intake, 2 studies assessed the add-on EPA effect, 1 study assessed the effect of dietary carbohydrate, and 2 cross-sectional studies assessed the association between carbohydrate intake and depression mood. One of these cross-sectional studies was specifically conducted among diabetics, but another study was conducted among healthy and diabetic patients. We also summarized the most important studies that were reviewed in the current paper in Table 1.

## 3. Dietary Carbohydrate

Carbohydrate intake seems to be an important factor in the management of depression and diabetes. Depressive symptoms are associated with lower consumption of vegetables and fruits [43], while overeating of high glycemic index (GI) foods is a routine coping in depressed and stressful patients [44–46]. The adverse effects of high GI on glycemic control and insulin resistance have been shown previously [47–49]. Therefore, in order to achieve a better glycemic control in diabetic patients, the management of carbohydrate intake may play a noteworthy role. Very few studies were conducted to assess either the effect of low or high consumption of carbohydrate or the effect of glycemic index and glycemic load (GL) on depressive symptoms in diabetic patients [36–38]. Carbohydrate consumption is associated with higher secretion of insulin which facilitates the transport of tryptophan in the brain and leads to higher synthesis of serotonin [46, 50, 51]. Thereby, higher GI and GL carbohydrate could potentially have more beneficial effects on depressive symptoms. However, two limitations remain which should be noted. First, higher glycemic index diets may be associated with lower diet quality and the lower intake of some key nutrients for depression such as magnesium, riboflavin, and dietary folate equivalents (DFE) [52]. Furthermore, higher GI index diets are mainly recognized with lower consumption of some nutrient-dense foods including vegetables, fruits, and fiber [53]. Poor diet quality and food choices, therefore, would be expected to adversely affect depressive symptoms. Another limitation is related to glycemic control. Although some studies showed that the amount of carbohydrate is the main concern rather than GI or GL in diabetic patient's diet [54, 55], others yet advice to prescribe a low-GI and GL diet for diabetics [47–49]. The entry of tryptophan into the brain is also related to its plasma concentration to the sum of the other large neutral amino acids (Trp/LNAAs ratio) (LNAAs: tyrosine, phenylalanine, leucine, isoleucine, valine, and methionine) [56]. High GI carbohydrates increase insulin levels and whereby thus stimulate the selective uptake of LNAAs by muscles and increase the Trp/NLAAs ratio [57]. It has been shown that elevated ratio of Trp/NLAAs which is achieved by carbohydrate consumption improves performance and mood under the stress [58]. 

A randomized clinical trial compared a low GI diet versus ADA diet in diabetic patients [36]. Both diets were similar in their carbohydrate, protein, saturated fatty acids (SFAs), and cholesterol content. Two prescribed diets contained 55% carbohydrate, but in low-GI diet participants were encouraged to consume low-GI carbohydrate, while in ADA diet, all carbohydrate foods were treated the same. After 12 months, participants in ADA group consumed significantly more carbohydrate but decreased GI. Although the GI did not differ significantly between two groups (80 in the ADA versus 76 in the low GI; *P* = 0.07), dietary GL was significantly lower in low-GI group compared with ADA after 6 months (97 versus 141; *P* = 0.02). Both diets showed significant improvement in metabolic markers after 12 months. In spite of a slight improvement in both dietary interventions, changes in depressive symptoms did not reach significant difference comparing the two interventions. 

Umegaki et al. [38] in a cross-sectional study on elderly diabetic patients showed that higher percentage of carbohydrate intake is positively associated with depression in women but not men. Another cross-sectional study on 976 homebound elders (30% of participants with type 2 diabetes) assessed the relationship between dietary GI and depression [37]. The findings of this study suggested that despite similar amount of carbohydrate consumption by both depressed and nondepressed, GI and serum insulin levels were significantly higher in depressed patients than nondepressed (*P* values = 0.003 and 0.05, resp.). Patients receiving antidepressant agents (specially selective serotonin reuptake inhibitors (SSRI)), had lower GI and GL compared with who did not (*P* = 0.002 and 0.03, resp.). The association between dietary GI, GL, the amount of consumed carbohydrate and depression are also inconsistent among nondiabetic patients [59–61]. Some reports showed that higher dietary GL decreases the risk of depression [59, 60], while others failed to reach such findings [61]. However, most of available studies have not linked dietary GI to depression [59, 61]. 

Totally, few studies were conducted among diabetic to assess the effect of dietary carbohydrate on depression symptoms. Available studies are inconsistent. While a clinical trial did not approve the effect of GI and GL on depression, observational studies showed significant association. Moreover, benefits and disadvantages of low-carbohydrate diets are still on debate and should be noted in the treatment of depression. While some clinical trials have advocated the beneficial effects of low carbohydrate diets (LCD) on metabolic markers and weight loss, their long term effects on psychological features have been poorly studied. Some short-term studies have shown that dietary composition could not significantly affect mood state [62–64]; however, there are some long-term evidences that showed adverse effects of LCD on mood state compared with low fat diet (LFD) among nondiabetic patients. Halyburton et al. showed that both LCD and LFD improve mood after 8 weeks [65], but following this study for 52 weeks dispute this finding. Brinkworth et al. showed that although decreased scores of depression and depression-dejection were stable over time for LFD, they returned toward baseline values for LCD after 52 weeks [66]. The adverse effects of LCD may be due to disturbed synthesis of serotonin or Brain-Derived Neurotrophic Factor (BDNF). More studies should be conducted to illustrate the beneficial and adverse effects of LCD on psychological function.

## 4. Dietary Protein

Renal disease, another complication of diabetes, has been widely claimed that is associated with depression [67–69]. Low protein diet (0.6–0.8 g/kg), the common treatment of chronic renal disease, has some beneficial effects on the progression of renal failure, inflammatory makers levels and coagulation proteins such as fibrinogen in nephropathic-diabetic patients [70–76]. 

A noteworthy aspect regarding the protein consumption and depression is related to its effect on Trp/LNAA. It has been shown that a high protein meal decreases the serum level of Trp while increases LNAAs and; therefore, decreases the Trp/LNAA ratio [56]. Hence, a low protein diet may have some beneficial effect on Trp/LNAAs, the Trp transmission into the brain and the synthesis of serotonin. However, a low protein diet provided lower amount of tryptophan which could interact with the beneficial effects of low protein diet. The effect of dietary protein intake on depression is unclear, and only two studies assessed the effects of low protein diet on depressive symptoms among diabetic patients in short and long term [39, 40].

In a randomized clinical trial, Ciarambino et al. [39] compared the effect of a 6-day/week low protein diet (6/7 LPD, with a day/week normal protein diet) with a 7-day/week one (7/7 LPD), after 4 weeks on young-old type 2 diabetic patients. LPD contained 0.8 g/kg protein which more than 65% of ingested protein was provided by high biological value. Before the beginning of study, all patients consumed a normal protein diet (1.2 g/kg) for at least 4 weeks. Normal protein diet contained 50% carbohydrate and 25% fat, while in low protein diet 35% of total energy intake was provided by fat (especially unsaturated fatty acids). The results of study showed that 7/7 LPD increased the symptoms of depression versus normal protein diet. In the other long term study [40], Ciarambino et al. followed up these patients for 30 months. At the end of study, 7/7 LPD significantly leads to more increment in the score of geriatric depression scale (GDS-15) rather than 6/7 LPD. The probable reason of such findings may be related to the lower content of thiamine, folate, iron, and Trp which induces cognitive changes consistent with depressive mood [77–79]. The proportion of dietary macronutrients must also be taken into account when interpreting the findings. On the other hand, the beneficial effects of normal protein diet and 6/7 LPD rather than 7/7 LPD might be due to the higher proportion of dietary fat, especially unsaturated fatty acids. We found very limited studies among depressed patients without diabetes which assessed the effect of different amount of protein consumption on depressive symptoms. The results of a 10-year follow-up study showed that higher protein intake is associated with lower but higher risk of depression among men and women, respectively [80]. One clinical trial could not find any significant difference between the effect of high-carbohydrate (En% Protein/Carbohydrate/Fat = 6/64/30) and high protein (En% Protein/Carbohydrate/Fat = 65/5/30) meals on psychological mood [81]. The results of our search did not show any study in relation to assess the effect of various types of protein on depression. More studies are necessary to illustrate the exact and clear effect of the amount and kind of dietary protein intake on depressive symptoms among diabetic (and nondiabetic) patients. 

## 5. Dietary Fat

Dietary fat intake has a strong role in determining stress oxidative and inflammation. Furthermore, both diabetes mellitus and depression are associated with lower n-3 polyunsaturated fatty acids (n-3 PUFA) concentrations [82–84] and disturbed lipid profile [85–87]. A hypothesis for explaining the increased prevalence of obesity, diabetes, and their comorbidities such as depression during last years, is related to increased intake of saturated fatty acid (SFA) and the ratio of n-6/n-3 PUFA [88]. The favorable effect of n-3 PUFA consumption has been approved either by epidemiological studies which assessed fish consumption and or by ones which assessed the red blood cell concentrations of n-3 PUFA [89–93]. However, the results of a meta analysis on 28 randomized and placebo controlled clinical trial showed that the efficacy of n-3 PUFA in depression is related to eicosapentaenoic acid (EPA) not docosahexaenoic acid (DHA) [94].

We did not find any epidemiological study regarding the association between n-3 PUFA consumption and depression among diabetic patients. We just find two clinical trials which assessed the effects of EPA consumption on depressive symptoms in diabetic [42, 95]. A randomized clinical trial by Bot et al. [42] did not confirm the beneficial effects of add-on supplementation of EPA (1 g/d) on depressive symptoms in diabetic patients, after 12 weeks. However, because of the strong link between EPA intake and some similar biological disturbances in diabetes and depression, such as hypothalamus-pituitaryadrenal (HPA)-axis hyperactivity [4] and one-carbon-cycle alterations [41], authors also assessed the effect of EPA on more biological markers [95]. They did not find any significant effects of EPA supplementation on oxidative stress, inflammatory, or one-carbon-cycle parameters compared with placebo. In this study, EPA consumption increased serum content of DHA, HDL-C and total cholesterol and decreased plasma concentration of arachidonic acid.

The relationship between other types of fatty acids and depression has been poorly studied. There is some evidence which may link depression and SFA as well as trans fatty acids (TFA) intake [96, 97]. First, the simultaneous increment in the prevalence of depression and dietary changes toward “western dietary pattern,” which is rich in SFA and TFA, might be one of the most important evidence. Second, there are similar pathogenesis for several chronic diseases and depression. The adverse effects of SFA and TFA on coronary heart diseases and diabetes mellitus have been well established. Because of similar pathophysiology for such chronic diseases and depression, the harmful effects of SFA and TFA could be also considered for depression. However, few studies assessed the association between SFA and TFA and depression. In a cohort study, Sánchez-Villegas et al. [96] showed that persons in the highest quintile of TFA had 48% increment in depression risk compared with first quintile, with a significant linear trend. They also showed that PUFA intake is inversely associated with depression, but n-3, n-6, and n-3 from fish and n-3 : n-6 were not associated with depression. In this study, a marginal significant inverse dose-response association was found for MUFA consumption. Most of observational and interventional studies among nondiabetic patients confirmed the health benefit of n-3 fatty acids on depressive symptoms [98–101]. However, the published literature is conflicting. For example, in one study, the inverse association between higher consumption of fish, DHA, EPA, and depression were observed only in boys but not girls [98]. Other study found a cross-sectional, but not longitudinal, association between depression and n-3 consumption [101]. The results of a clinical trial did not confirm the beneficial effects of n-3 supplementation on amelioration of depressive symptoms [102]. There are such discrepancies for other fatty acids. Some investigators found a link between the erythrocytes concentration of TFA, short chain SFA, short chain MUF (lower than 18 carbons), and depression [84, 103], while other study showed higher concentration of SFA decreases the risk of depression [103]. Assies et al. found lower concentration of long chain SFA, MUFA and PUFA in the erythrocyte of patients with depression in comparison with nondepressed [84]. 

The beneficial effects of MUFA, and PUFA intake on depression might be related to their association with inflammation. Higher levels of inflammatory markers in depressed persons may reduce serum tryptophan level and impair neurotransmitter metabolism by inhibition of the gene expression of Brain-Derived Neurotrophic Factor (BDNF) [104, 105]. Lower level of BDNF has been shown in depressed patients [106]. Therefore, beneficial subtypes of fatty acids could improve depressive symptoms by modulating serum levels of inflammatory markers. Furthermore, endothelium is the source of BDNF synthesis and secretion [107], and TFA promote endothelial dysfunction [108] and therefore consequently lead to lower level of BDNF. Beside the types of fatty acids, the amount of fat consumption is another determinant of depression. It has been shown that low fat diets could adversely affect mood [109, 110]. However, to the best of our knowledge, there is no study assessing the effect of dietary fat amount on depressive symptoms among diabetic patients.

## 6. Dietary Patterns

Depression is associated with unhealthy food choices. Several studies showed higher consumption of unhealthy foods and lower consumption of fruits and vegetables in depressed mood [111]. Although dietary patterns and diet quality are a novel area of attention in the lifestyle-mental health research field, there is no study in this field among diabetic patients. The results of Whitehall II prospective cohort study showed that “whole food” pattern (rich in vegetables, fruits, and fish) was inversely associated with depression, while “processed food” pattern (rich in sweetened desserts, fried food, processed meat, refined grains, and high-fat dairy products) showed a direct association with depression in middle-aged population [112]. In this population the prevalence of diabetes mellitus was significantly higher among depressed persons compared with nondepressed ones (5% versus 2.5%; *P* = 0.003). However, another study among Japanese did not show any significant association between depression and various dietary patterns (including “Healthy,” “Western,” “Bread and confectionery,” and “Alcohol and accompanying”) [113]. Furthermore, in this study, various dietary determinants of depression such as energy intake, vitamin B2, B6, B12, and n-3 PUFA did not significantly differ between depressed and nondepressed. In the GAZEL cohort study, after 1-year followup [114], Le Port et al. reported that men who were in the highest quartile of low-fat, western, high snack, and high fat-sweet diets and women who were in the highest quartile of low-fat and high snack were more likely to be depressed versus the lowest quartile. Conversely, the highest quartile of healthy and traditional dietary patterns were associated with lower odds ratio for depression among women compared with the lowest quartile. In this study traditional diet was loaded on fish and fruit consumption and healthy diet was loaded on vegetables consumption. Another finding from the SUN cohort study demonstrated an inverse relationship between the adherence to Mediterranean diet and depression, while consumption of fast foods and commercial baked goods increased the risk of depression among Spanish [115, 116].

Although the findings of Sugawara' study [113] did not confirm the association between dietary pattern and depression, many studies showed that diet quality is a protective factor against depression [117, 118]. Interestingly, the serum concentration of folate, as a determinant of depression, is a marker of diet quality [119]. However, many observational studies reported low diet quality among diabetic patients [120, 121]. Additionally, because of the cumulative and synergic effect of nutrients from different dietary sources compared with the effect of one isolated nutrient, assessing the whole diet is preferred to study various nutrients, separately. 

## 7. Conclusion

While depression is a common disorder among diabetic patients, which could affect the success of treatment, very limited studies were conducted among diabetic patients to treat depression. In conclusion, dietary recommendations for depression are the same for metabolic syndrome, because of the similar pathophysiology [122]. SFA, TFA, low fat diet, lower quality diets, and unhealthy dietary patterns such as western diet were associated with more depression symptoms while n-3 PUFA, MUFA, Mediterranean dietary pattern and other healthy dietary pattern which heavily characterized by fruit, vegetables, and fish were inversely associated with depressive symptoms. In present study we focused on the role of different macronutrients in treatment of depression. However, isolation of the effect of one component of nutrients from others on the endpoints is difficult. On the other hand, decreasing the proportion of a nutrient is inevitably associated with increasing the proportion of other nutrients. Hence, it is difficult to attribute outcomes to the decreased nutrient or increased one. More studies are needed to clarify the effects of replacing various nutrients (in different types, for example, vegetable or animal proteins, simple or complex carbohydrates, saturated or unsaturated fat and their dietary sources) with each other on mood and depressive symptoms. Moreover, because of various comorbidities in diabetic patients such as renal failure, neuropathy, and coronary heart diseases, the appropriate range of various macronutrients as well as the proper type of each macronutrient should be clearly defined. 

## Figures and Tables

**Table 1:** A summary of the most important papers which were reviewed in the current paper.

Reference/year	Subjects/country	The intervention and control diets	The duration of intervention	Depression assessment	findings
Ma et al. [36], 2008	40 individuals with poorly controlled type 2 diabetes, USA	Parallel and randomized clinical trial/Low GI versus ADA dietary education	12 months	Center for Epidemiological Studies-Depression Scale (CES-D)	In spite of a slight improvement in both dietary interventions, changes in depressive symptoms did not reach significant difference between 2 interventions

Mwamburi et al. [37], 2011	976 homebound elders (30% of participants with type 2 diabetes), USA	Cross-sectional study	—	Center for Epidemiological Studies-Depression Scale (CES-D)	Depressed patients had higher dietary GI than non-depressed.

Umegaki et al. [38], 2009	653 elderly diabetic patients, Japan	Cross-sectional study	—	GDS-15 scores of 6 and higher	Positive association between higher percentage of carbohydrate intake and depression in women but not men

Ciarambino et al. [39], 2011	52-years-old type 2 diabetic patients with renal failure, Italy	At first a normal protein diet (1.2 g/kg/d), then randomization for consuming either an LPD (0.8 g/kg/d) for 7 d a wk (LPD 7/7) or for 6 d a wk (LPD 6/7)	4 weeks	Geriatric Depression Scale (GDS-15) and Beck Depression Inventory (BDI)	7/7 LPD increased the symptoms of depression versus normal protein diet.

Ciarambino et al. [40], 2012	38 elderly Type 2 diabetic patients with CRD (Stage 3–4), Italy	After 4 weeks on a normal protein diet (1.0 g/kg/d), participants were assigned for an LPD (0.7 g/kg/day), either 7 days a week (LPD 7/7) or 6 days a week (LPD 6/7).	30 months	Geriatric Depression Scale (GDS-15)	7/7 LPD significantly leads to more increment in the score of geriatric depression scale (GDS-15) rather than 6/7 LPD.

Stanger et al. [41], 2009	25 DM (type 1 or 2) and MDD, who used their current antidepressant medication for at least two months, Netherlands	Randomized, double-blind, placebo-controlled trial/comparing add-on ethyl-EPA-supplementation (1 g/d) to placebo	12 weeks	DSM-IV	No beneficial effects of EPA on depressive symptoms

Bot et al. [42], 2010	25 DM and MDD patients, who were already using antidepressant medication, Netherlands	Randomized, placebo-controlled, double-blind, parallel-group/comparing add-on ethyl-EPA-supplementation (1 g/d) to placebo	12 weeks	DSM-IV	No beneficial effects of EPA on depressive symptoms
